# Tuberculosis drug resistance in Bamako, Mali, from 2006 to 2014

**DOI:** 10.1186/s12879-016-2060-7

**Published:** 2016-11-28

**Authors:** B. Diarra, D. Goita, S. Tounkara, M. Sanogo, B. Baya, A. C. G. Togo, M. Maiga, Y. S. Sarro, A. Kone, B. Kone, O. M’Baye, N. Coulibaly, H. Kassambara, A. Cisse, M. Belson, M. A. Polis, J. Otu, F. Gehre, M. Antonio, S. Dao, S. Siddiqui, R. L. Murphy, B. C. de Jong, S. Diallo

**Affiliations:** 1 SEREFO Program, University of Sciences, Techniques and Technologies of Bamako (USTTB), Bamako, Mali; 2 Laboratoire National de Référence des Mycobactéries (LNR), Institut National de Recherche en Santé publique (INRSP), Bamako, Mali; 3 CCRB, Division of Clinical Research, National Institute of Allergy and Infectious Diseases, Bethesda, MD USA; 4 Vaccines and Immunity Theme, Atlantic Boulevard, Medical Research Council (MRC), Fajara, Banjul, The Gambia; 5 Department of Biomedical Sciences, Institute of Tropical Medicine, Antwerp, Belgium; 6 Faculty of Infectious and Tropical Diseases, London School of Hygiene & Tropical Medicine, London, UK; 7 Microbiology and Infection Unit, Warwick Medical School, University of Warwick, Coventry, UK; 8 Global Health, Northwestern University, Chicago, IL USA

**Keywords:** Tuberculosis Drug resistance, Bamako, Mali

## Abstract

**Background:**

Although Drug resistance tuberculosis is not a new phenomenon, Mali remains one of the “blank” countries without systematic data.

**Methods:**

Between 2006 and 2014, we enrolled pulmonary TB patients from local TB diagnostics centers and a university referral hospital in several observational cohort studies. These consecutive patients had first line drug susceptibility testing (DST) performed on their isolates. A subset of MDR was subsequently tested for second line drug resistance.

**Results:**

A total of 1186 mycobacterial cultures were performed on samples from 522 patients, including 1105 sputa and 81 blood samples, yielding one or more *Mycobacterium tuberculosis* complex (Mtbc) positive cultures for 343 patients. Phenotypic DST was performed on 337 (98.3%) unique Mtbc isolates, of which 127 (37.7%) were resistant to at least one drug, including 75 (22.3%) with multidrug resistance (MDR). The overall prevalence of MDR-TB was 3.4% among new patients and 66.3% among retreatment patients. Second line DST was available for 38 (50.7%) of MDR patients and seven (18.4%) had resistance to either fluoroquinolones or second-line injectable drugs.

**Conclusion:**

The drug resistance levels, including MDR, found in this study are relatively high, likely related to the selected referral population. While worrisome, the numbers remained stable over the study period. These findings prompt a nationwide drug resistance survey, as well as continuous surveillance of all retreatment patients, which will provide more accurate results on countrywide drug resistance rates and ensure that MDR patients access appropriate second line treatment.

**Electronic supplementary material:**

The online version of this article (doi:10.1186/s12879-016-2060-7) contains supplementary material, which is available to authorized users.

## Background

Tuberculosis (TB) continues to be a global public health concern with an estimated 9.6 million incident cases, and 1.5 million deaths worldwide in 2014 [1]. The global estimate in 2014 of multidrug-resistant TB (MDR-TB) was 480 000 new patients worldwide including cases of primary and acquired MDR-TB [1], of whom 190, 000 died [1]. Drug resistant tuberculosis (DR-TB) continues to threaten global tuberculosis control, resulting from either primary infection with resistant bacteria or from acquired resistance. MDR-TB is results from bacteria that are resistant to at least isoniazid and rifampicin, the most effective anti-TB drugs available. Extensively drug-resistant TB (XDR-TB) strains are additionally resistant to any fluoroquinolone and any of the second-line anti-TB injectable drugs (amikacin, kanamycin or capreomycin).

Mali, a large country with limited resources, reported in 2014 9800 new TB patients, including 40 MDR-TB patients [1]. The world health organization (WHO) estimates the number of MDR cases in Mali to be 130 among notified pulmonary TB patients, based on sub-national data assumptions, as no formal drug resistance survey has been conducted to date [1]. In Mali, individual patient drug susceptibility testing (DST) is not universally accessible, with only few laboratories capable of performing such activity: the national TB reference laboratory (NRL) and the biosafety level 3 (BSL-3) laboratory of SEREFO (HIV and TB research and training center of the University of Sciences, Techniques and Technologies of Bamako (USTTB) conduct phenotypic testing, while the private Charles Mérieux Institute laboratory has implemented the Hain *MTBDRplus* Line Probe Assay to detect resistance mutations to isoniazid and rifampicin. Thus, DST is restricted at the NRL to retreatment patients, such as those who fail, lost to follow up, or relapse after completion of the standardized 6 months first line therapy and no systematic testing for drug resistance in new TB patients has been performed to date in Mali.

However, at SEREFO, DST has been conducted since 2006 for each new and retreatment patient enrolled in research protocols. In addition, per national guidelines, all suspected DR-TB patients are referred to the NRL for culture and DST, and MDR patients are referred to the TB unit of the university teaching hospital of Point-G, Bamako, for further assessment and second line therapy. Thus, MDR patients included in SEREFO’s protocols have had parallel culture and DST conducted as external quality control (QC) for the NRL, essentially monitoring consecutive retreatment patients in Mali since 2006.

Although DR-TB is not a new phenomenon, Mali remains one of the ‘blank’ countries without systematic data in the WHO reports, in contrast to other countries in the region, such as Benin and Senegal [2, 3]. In the absence of a nationwide drug resistance survey, we present the evolution of tuberculosis drug resistance among new and retreatment patients, and reviewed the demographic features of patients, drugs resistance profiles and disease characteristics of MDR-TB in Mali over the 9 year period from 2006 to 2014.

## Methods

### Study design and setting

Between 2006 and 2014, we conducted multiple descriptive cohort studies by enrolling pulmonary TB patients from local reference centers and the University Teaching Hospital at Point G, in Bamako, Mali, whose mycobacterial isolates had been cryopreserved. In the present cross-sectional analysis, we pooled baseline drug susceptibility testing by treatment history for all patients who enrolled in any of the studies, and referred a subset of cryopreserved isolates to MRC Gambia for additional phenotypic resistance testing.

Mali is a landlocked West African country with a size of 1,241,248 km^2^ and population of approximately 14.5 million people [4]. Bamako, the capital city, has a population of approximately two million people and is divided into six urban districts, with each district having a health referral center, where TB diagnostic and treatment services are available. The university teaching hospital at point-g is located in district three and serves as the main tuberculosis reference center. In 2014 alone, more than one third of the total TB patients in Mali were managed in Bamako, and our study population, which represented more than 80% of the notified Malian retreatment cases, were all referred to point-g hospital for further investigations and appropriate treatment [5].

### Study population

Suspected pulmonary TB patients were screened at the study sites based on smear positive result of either Ziehl Neelsen (ZN) or Auramine/Rhodamine (A/R) staining and consenting participants were enrolled into the studies (for in- and exclusion criteria see Additional file 1: Table S1). Only TB patients who had *Mycobacterium tuberculosis* complex (Mtbc) disease confirmed by culture were included in the final analysis (Additional file 1: Table S1).

Patients were classified based on their TB treatment history as new patients, who had received TB treatment for less than 4 weeks, and as relapse and/or treatment failure patients, who had received TB treatment for more than 1 month according to protocol inclusion criteria. However, in 2009 and 2010, there were few studies running, and most patients screened at that period originated from the programmatic surveillance of retreatment patients who were more likely to be drug resistant patients. Patients were treated in accordance with national guidelines of the TB program in Mali [6]. New patients received a fixed dose combination of 2 months of rifampicin (R), isoniazid (H), pyrazinamide (Z) and ethambutol (E) and 4 months of (R) and (H) (2RHZE/4RH; Cat 1), while re-treatment patients received 2 months of (R), (H), (Z), (E) and streptomycin (S), followed by 1 month with the same combination but without (S) and 5 months of (R), (H), and (E) (2RHZES/1RHZE/5RHE; Cat. 2). Patients clinically suspected of having MDR disease (those who failed Cat. 2 regimen) or confirmed MDR-TB patients underwent a 6 month course of kanamycin (K), ofloxacin (O), ethionamide (Et) and (Z) during the 6 months inpatients period, and followed by 15 months of the same combination without kanamycin (6KOEtZ/15OEtZ) [6]. Per national guidelines, patients with confirmed MDR were checked on a monthly basis for treatment response by physical examination and sample collection for microscopy and culture. During the study period, programmatic treatment outcome was monitored for confirmed MDR patients several months after leaving the hospital while still on treatment, as well as several months after treatment.

### Laboratory tests

Pre-enrollment sputum smear microscopy by ZN or direct fluorescent smear microscopy using Auramine/Rhodamine (FM) at local reference centers was followed by indirect FM and culture at SEREFO laboratory, which is certified by the college of American pathologists (CAP) as external quality control. Confirmed Mtbc isolates underwent drug susceptibility testing (DST). In the context of a regional collaboration, subcultures had DST repeated for first line drugs, plus second line DST for a randomly selected MDR isolates, at the Medical Research Council (MRC) Unit, The Gambia.

### Culture and susceptibility testing

Sputum specimens were digested and decontaminated using the standard N-Acetyl-L-Cysteine/4% NaOH solution, concentrated by centrifugation (4500 rpm) and inoculated on both liquid (*Mycobacterium* Growth Incubator Tube (BBL™ MGIT™ Becton Dickinson, Sparks MD, USA)), and solid (Middlebrook 7H11 Agar and Selective 7H11 Agar) media. Simultaneously, an aliquot of concentrated specimen was prepared for indirect commercial Auramine-Rhodamine staining (BBL™ Becton Dickinson, Sparks MD, USA). Speciation of positive mycobacterial cultures was based on acid fast bacilli (AFB) positivity in fluorescent microscopy (FM) and colony morphology on solid medium, and was confirmed by nucleic acid probes (AccuProbe® GenProbe, San Diego, CA, USA). Indirect drug susceptibility testing was performed on Mtbc isolates using MGIT AST/SIRE System (Becton Dickinson, Sparks, MD, USA) and the results were interpreted based on the United States of America (USA) Center for Disease Control (CDC) recommendations for critical drug concentrations [7]. Moreover, indirect DST by MGIT using second line drugs, ofloxacin (OFX, 2 μg/mL), kanamycin (KAN, 6.0 μg/mL), capreomycin (CAP, 2 · 5 μg/mL, Sigma-Aldrich, St. Louis, Mo), USA was performed on randomly selected MDR isolates at MRC, The Gambia. A subset of MDR isolates identified from the repeated first line drug susceptibility testing at the MRC underwent second line drug susceptibility testing.

### HIV testing

The human immunodeficiency virus (HIV) serology status was determined using the following algorithm: first, rapid testing was done with Determine® (HIV-1/2, Abbott Laboratories, Matsudo-Shi, Chiba, Japan) for all participants, followed by an ELISA test for all patients (Genscreen Ag-Ac UltraHIV-1/2 version 2 Assay, Bio-Rad Laboratories, Marnes, France), and any positive ELISA test was confirmed by Western Blot test (New Lav Blot I/II (New Lav Blot I and II, Bio-Rad Laboratories, Marnes, France).

### Data & statistical analysis

Univariate and multivariate analyses were conducted with demographic and clinical variables to identify predictors of rifampicin resistance and MDR-TB. Results were considered significant when p-values were less than 0.05. Analyses were performed using SAS version 9.1 (SAS Institute, Cary, NC) and Epi info version 3.5.3 (CDC, Atlanta, USA).

## Results

**Fig. 1:**
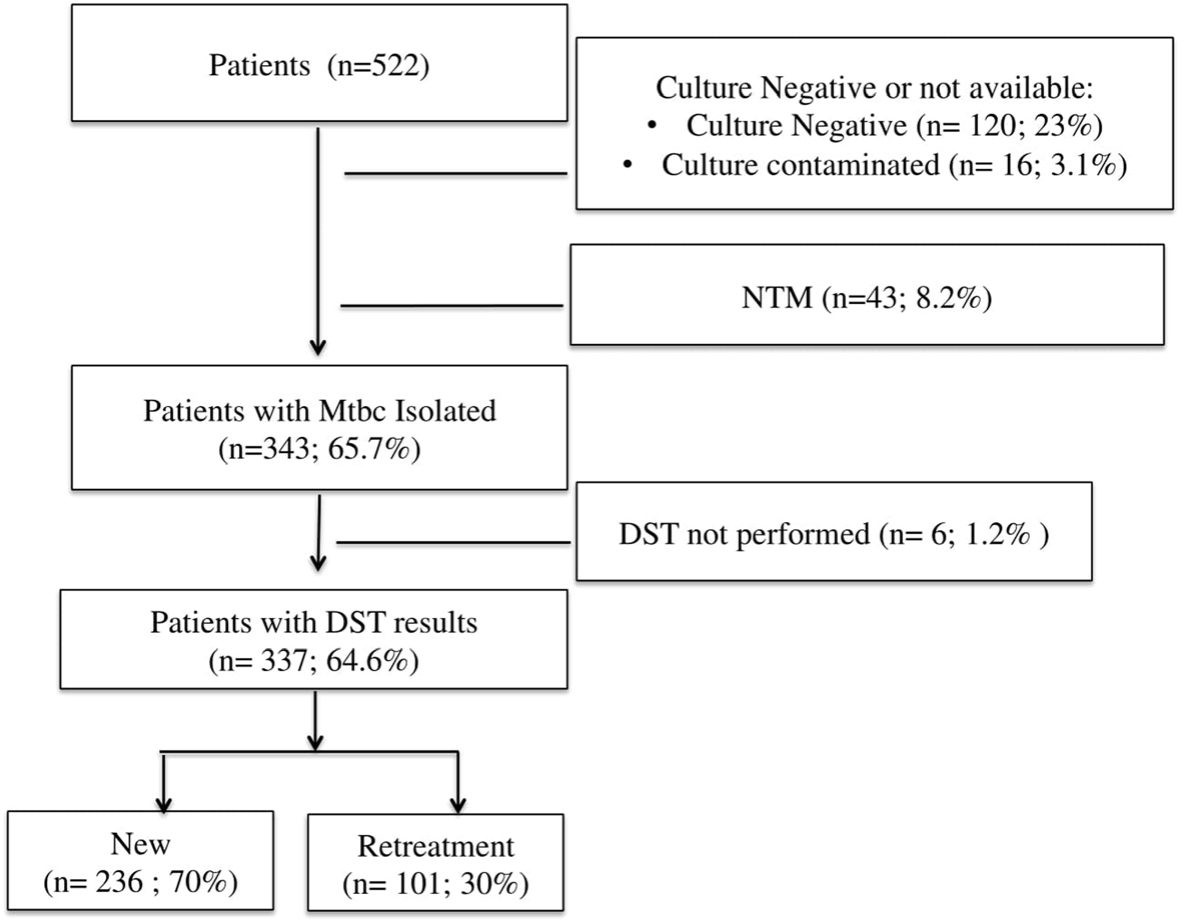
Flow chart of patients included in the study. Mtbc*. = Mycobacterium tuberculosis* complex, NTM = Non-tuberculous Mycobacteria, DST = Phenotypic drug susceptibility Testing

A total of 1186 mycobacterial cultures were performed in the SEREFO BSL-3 laboratory during the 9 year period, on samples from 522 patients, including 1105 sputa and 81 blood samples. Of these patients, 343 (65.7%) grew Mtbc, 43 (8.2%) grew non-tuberculous mycobacteria (NTM), 120 (23%) had a negative culture, and 16 (3.1%) were contaminated (Fig. 1). Among the 522 patients, 395 (75.7%) were male and 69 (13.2%) were HIV positive. Of the 343 patients in whom tuberculosis was culture confirmed, 242 (70.6%) were new patients, and 101 (29.4%) were previously treated patients. The majority (61.8%) of the patients had been enrolled in studies including both new and retreatment patients, and the remainder in studies only including new TB patients (Additional file 1: Table S1). Phenotypic drug susceptibility testing (DST) was performed on 337 (98.3%) of the Mtbc isolates (Tables 1 and 2).

**Table 1:** General profile of phenotypic drug susceptibility testing profile per year

Phenotypic drug susceptibility testing profile	2006	2007	2008	2009	2010	2011	2012	2013	2014	Total
Total number of isolates tested	18	53	33	15	10	29	53	87	39	337
Pan susceptible	18	37	22	2	1	16	34	63	17	210
S	0	1	3	0	0	0	2	8	2	16
I	0	0	1	1	0	2	4	2	2	12
R	0	1	0	0	0	0	0	0	1	2
E	0	0	0	0	0	0	1	0	0	1
S, I	0	1	2	2	0	0	1	5	5	16
S, I, E	0	1	0	0	0	0	1	1	0	3
S, R	0	0	0	0	0	0	0	0	1	1
S, R, E	0	0	0	0	0	0	0	0	1	1
S, I, R	0	2	0	0	2	0	0	0	1	5
I, R, E	0	2	1	1	0	1	0	0	0	5
I, R	0	1	1	0	0	3	1	0	4	10
S, I, R, E	0	7	3	9	7	7	9	8	5	55
Total # of MDR-TB per Year	0	12	5	10	9	11	10	8	10	75
% of MDR-TB per Year	0.00	22.6	15.2	66.7	90.0	37.9	18.9	9.2	25.6	22.3

*S* streptomycin, *I* isoniazid, *R* rifampicin, *E* ethambutol, *MDR-TB* multidrug resistance (resistance to at least isoniazid and rifampicin)

**Table 2:** Patterns of resistance to first-line anti-tuberculosis drugs

Type of resistance	New patients (*n* = 236) n (%)	Previously treated patients (*n* = 101) n (%)
Susceptible to all drugs	193 (81.8)	17 (16.8)
Any resistance		
S	25 (10.6)	68 (67.3)
I	25 (10.6)	76 (75.2)
R	10 (4.2)	69 (68.3)
E	7 (3.0)	52 (51.5)
Monoresistance		
S	13 (5.6)	3 (3.0)
I	10 (4.2)	2 (2.0)
R	1 (0.4)	1 (1.0)
E	0 (0)	1 (1.0)
Total	24 (10.2)	7 (6.9)
I + R resistant (MDR-TB)		
SIR	0 (0)	5 (5.0)
IR	3 (1.3)	7 (6.9)
IRE	1 (0.4)	4 (4.0)
SIRE	4 (1.7)	51 (50.5)
Total	8 (3.4)	67 (66.3)
Other resistance^a^		
SI	8 (3.4)	8 (7.9)
SIE	1(0.4)	2 (2.0)
SR	0 (0)	1 (1.0)
SRE	1 (0.4)	0 (0)
Total	10 (4.2)	11 (10.9)

*S* streptomycin, *I* isoniazid, *R* rifampicin, *E* ethambutol, *MDR* multidrug-resistant, *TB* tuberculosis

^a^Defined as resistance to more than one drug, except for multidrug resistance

### Prevalence of first-line drug resistance in our study population

**Fig. 2:**
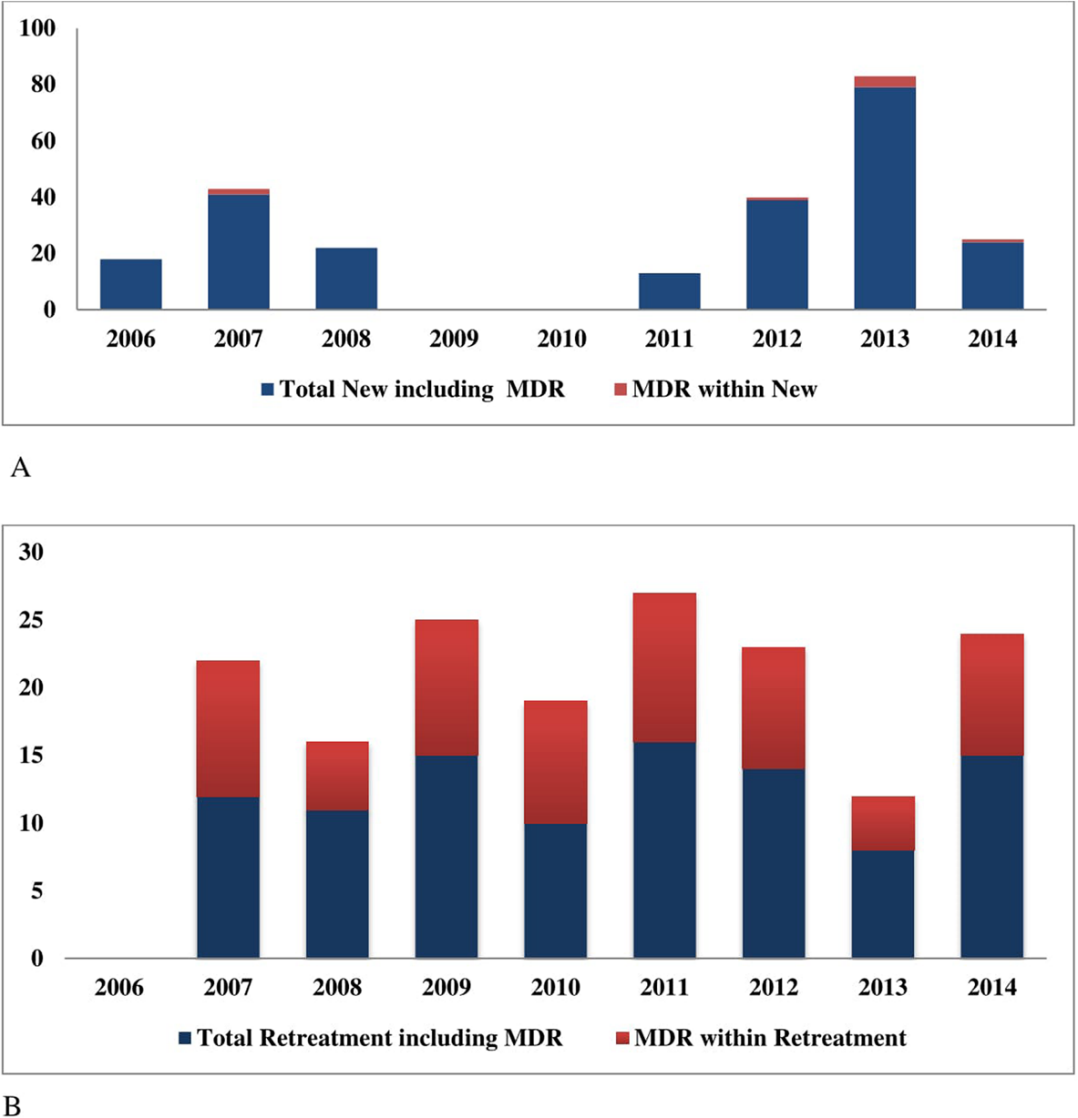
The evolution of Tuberculosis drug resistance within the 9-year period. **a** The evolution of MDR-TB patients from newly infected patients, and (**b**) The evolution of MDR-TB from retreatment patients. The total number in each case includes the MDR cases. MDR-TB: multidrug resistant Tuberculosis; DST, drug susceptibility testing. While the absolute number of MDR patients remains relatively stable, the proportion varies widely because recruitment rates of new patients were variable. In 2006 and 2009, no MDR-TB patient was identified within recruited patients, and in 2010, no new TB patients were recruited and all retreatment patients tested were MDR

Among the 337 patients whose isolates underwent DST, 127 (37.7%) had resistance to at least one drug, including 75 (22.3%) multidrug resistant (MDR) cases. We observed two (0.6%) rifampicin mono-resistant isolates, and 12 (3.6%) isoniazid mono-resistant isolates (Table 1). The prevalence of MDR-TB was 3.4% among new patients and 66.7% among retreatment patients (Table 2). Of the 75 MDR-TB patients, eight (10.7%) were identified among new, and 67 (89.3%) were identified among retreatment (Fig. 2, a and b). Indirect FM was positive in 176 (84.6%) of patients without resistance, and in 97 (78.9%) of the patients with resistance (Table not shown). The prevalence of polyresistance (defined as resistance to more than one drug, except for MDR) was 4.2% among new TB patients, and 10.9% among previously treated patients (Table 2). Retesting of first line DST results on 208 randomly selected isolates at MRC showed 99% concordance for isoniazid, 98% for rifampicin, 92% for ethambutol, and 91% for streptomycin (Table not shown).

Demographic characteristics did not differ significantly between patients with and without rifampicin resistance, except for a higher proportion of patients with rifampicin resistance with lower smear grades (AFB smear negative and/or 1+/2+, compared with 3+, *p < 0.01*), and, as expected, a higher rate of MDR-TB among previously treated patients (*p* = <0.01*)* (Table 3).

**Table 3:** Demographic characteristics and treatment antecedent of patients with- and without rifampicin resistance

Characteristics	Patients with rifampicin resistance (N = 79) n (%)	Patients without rifampicin resistance (N = 258) n (%)	*P* value
Age (year) 0–14	0 (0)	2 (0.7)	*-----*
15–24	14 (17.7)	62 (24)	0.24
25–34	26 (32.9)	92 (35.6)	0.65
35–44	19 (24)	41 (15.9)	0.09
45–54	9 (11.4)	33 (12.8)	0.74
≥ 55	7 (8.9)	22 (8.5)	0.92
Female	16 (20.2)	63 (24.4)	0.44
HIV+	6 (7.6)	36 (13.9)	*0.13*
Previously Treated	69 (87.3)	32 (12.4)	*<0.01*
AFB Negative	8 (10.1)	6 (2.3)	*<0.01*
1+ or 2+	15 (18.9)	21 (8.1)	*<0.01*
3+	55 (69.6)	222 (86)	*<0.01*

*AFB* acid fast bacilli, *1+* smear AFB positive at 1+, *2+* smear AFB positive at 2+, *3+* smear AFB positive at 3+

### Prevalence of second-line drug resistance

Second line antimycobacterial susceptibility testing at MRC Unit, The Gambia including ofloxacin, kanamycin, and capreomycin, was available for 38 (50.7%) of the MDR-TB patients. While no XDR-TB was identified, the prevalence of resistance to either a fluoroquinolone or second-line injectable drug, but not both was 18.4% (Table 4). The proportion of single second-line drug resistance was 1/38 for ofloxacin, 1/38 for kanamycin, and 5/38 for capreomycin (Table 4).

**Table 4:** Second-line drug testing results on MDR-TB isolates

	Ofloxacin	Injectable drugs		Total Resistance to either Fluoroquinolone or injectable drugs N (%)
	Ofloxacin R, n (%)	Kanamycin R, n (%)	Capreomycin R, n (%)	
New MDR patients (*n* = 5)	0 (0)	0 (0)	0	0 (0)
Previously treated MDR patients (*n* = 33)	1 (3)	1 (3)	5 (15.2)	7 (21.2)
Total	1	1	5	7 (18.4)

*R* resistant, *n* number of strains with resistance to drug tested, *N* total number of strains with resistance

### Outcome of MDR-TB patients

Programmatic outcome measurements were available for 45 (60%) of the MDR-TB patients, of whom 13 (30%) died, 15 (33%) were lost to follow up, and only 17 (37%) experienced treatment success (treatment completion/cure).

## Discussion

In resource limited settings with a high incidence of tuberculosis, such as Mali, estimates of drug resistance are incomplete. We present, here a report on TB drug resistance profiles in Mali, over a 9 year period, based on baseline DST results from new and retreatment patients enrolled at the TB clinic of the university teaching hospital of point-g. While there was heterogeneity among the inclusion criteria of the different protocols (Additional file 1: Table S1), with some protocols excluding retreatment TB patients, we do not expect this to have affected the prevalence of resistance stratified within the new vs retreatment patient categories. However, the population referred to this clinic and its collaborating SEREFO BSL-3 laboratory is likely enriched for retreatment patients who had received multiple TB treatment episodes and thus likely overestimates the overall prevalence of DR-TB in Mali (Additional file 1: Table S1). Among the 38 (50.7%) of MDR-TB patients whose isolate was tested for second line drugs, we identified additional resistance in seven (18.4%), which was stable over time.

The present study was limited to Bamako city, the capital and largest city in Mali, where more than one third of the country’s TB patients are diagnosed [5]. A larger sample size and representative sampling from all of the different regions of Mali is therefore essential to obtain a true country profile of TB drug resistance. The planning and conduct of a true nationwide survey is however complicated by current terrorism activities and resulting instability in the north of the country. Despite its limitations, our study showed that the number of MDR-TB patients remained stable during the study period, (Table 1, Fig. 2), as the largest variation in the activity of study protocols affected recruitment of new TB patients, who had a low MDR-TB prevalence. Consequently, for 2009 and 2010, when few studies were recruiting, most patients screened originated from the programmatic surveillance of retreatment patients who were more likely to be drug resistant patients (Fig. 2). If the prevalence of MDR-TB of 3.4% among new patients, and 66.7% in previously treated patients were reflective of levels in the general population, Mali could be classified as having high levels of TB drug resistance, higher than many other West African countries such as Benin and Senegal where the MDR-TB prevalence was respectively 1.6 and 2.1% among newly diagnosed patients in 2007 [2, 3, 8]. In contrast, our data is similar to those from Rwanda in 2007 (3.0% MDR among new patients), and to the WHO global estimates of 3.6% of MDR-TB among new patients [1, 3], although MDR-TB among previously treated patients worldwide was lower at 20% [1]. We found that approximately 7% of newly infected patients had resistance to at least one drug. This is relatively high as the rate of primary drug resistance can be used to determine the efficacy of long-term tuberculosis treatment in the community [9]. As observed in many other studies, we found that MDR-TB is more common in previously treated patients, which group is likely enriched for patients failing multiple episodes of treatment (Fig. 2). This high rate has implications for infection control, as recent mathematical models confirm that most rifampicin resistant TB arises due to transmission of a rifampicin resistant isolate rather than acquired drug resistance [10]. Stratified analysis of patients with rifampicin resistance vs. without rifampicin resistance found that patients with rifampicin resistance were more likely paucy bacillary compared to the group without rifampicin resistance (Table 3). We did not find a relation between MDR-TB and HIV infection. The HIV seroprevalence we identified among TB patients in our studies (12.7%) was more than 11 times greater than in the general population (1.1%)[11, 12], reinforcing the need for screening all TB patients for HIV.

The outcome of MDR-TB treatment was poor for those patients with results available, showing a high mortality and a high loss to follow up. This may be due to the poor efficacy and tolerability of the WHO recommended treatment of MDR-TB. Fortunately, implementation of an improved regimen of 9 months duration has now been approved by the national ethics committee [13].

We found no XDR-TB in our study population although 18.4% of the MDR isolates had resistance to either fluoroquinolone or second line injectable drugs, and thus, the efficacy of second line treatment of these strains may be impaired, due to resistance to either fluoroquinolones or injectable drugs. All the MDR isolates with resistance to either fluoroquinolone or second-line injectable drugs were from previously treated patients and none was identified among MDR isolates from newly infected patients (Table 4). Among the second line drugs, the prevalence of resistance to capreomycin was the highest, which may impair effective MDR TB therapy, and thus result in poor outcome. The mono-resistance to kanamycin or capreomycin seen in our study is uncommon, as there usually is cross resistance to both drugs. Either this constitutes a clonal infection, to be confirmed by genotyping, or false resistance to capreomycin should be taken into consideration.

Early detection of rifampicin resistant TB is clearly a high priority for Mali, by increasing the coverage of surveillance among retreatment patients, supported by the use of the Xpert MTB/RIF at the NRL. Priorities for the management of drug resistant TB patients in Mali include the implementation of molecular and/or phenotypic second line DST, including minimal inhibiting concentrations (MICs) for ofloxacin or a different fluoroquinolone, to identify those patients who would benefit from high dose fluoroquinolone therapy. The advent of a shorter and more effective MDR regimen gives hope to improve on the dismal outcome of patients who were diagnosed with MDR in the past. This should be accompanied by improved methods to follow treatment efficacy in MDR patients, as monthly cultures are often not available and associated with long delays and high rates of contamination.

## Conclusion

The drug resistance levels, including the number of MDR-TB patients, found in this study population in Mali over the past 9 years are relatively high, likely related to the selected referral population. While worrisome, the numbers appeared stable over the study period. As culture and DST are only available in Bamako, the nationwide surveillance of retreatment patients, as recommended by WHO, would benefit from wider implementation of the GeneXpert MTB/RIF assay. In the absence of a formal nationwide drug resistance survey, such continuous surveillance will provide more accurate results on nationwide drug resistance rates. Second line drug resistance testing, currently not available in Mali, is another priority, to allow MDR-TB patients to access appropriate treatment regimens.

## Additional file

Additional file 1: Table S1. Summary of Studies Protocol’s inclusion and exclusion criteria. (DOC 34 kb)

## Abbreviations

A/R: Auramine/Rhodamine
BD: Becton Dickinson
CDC: Center for Disease Control
CI: Confidence interval
DR-TB: Drug resistant-tuberculosis
DST: Drug susceptibility testing
E: Ethambutol
Et: Ethionamide
H: Isoniazid
HIV: Human immunodeficiency virus
IRB: Institutional Review Board
K: Kanamycin
MDR: Multidrug resistant
MGIT: Mycobacterium growth incubator tube
MRC: Medical Research Council
Mtbc: *Mycobacterium tuberculosis* complex
NIAID/NIH: National Institutes of Allergy and Infectious Diseases, National Institutes of Health
NRL: National reference laboratory
NTM: Non-tuberculous mycobacteria
O: Ofloxacin
QC: Quality control
R: Rifampicin
S: Streptomycin
TB: Tuberculosis
USA: United Sates of America
USTTB: University of Sciences, Techniques and Technologies of Bamako
WHO: World Health Organization
XDR: Extensively drug resistance
Z: Pyrazinamide
ZN: Ziehl Neelsen
