# Supplementary material for: Tuberculosis drug resistance in Bamako, Mali, from 2006 to 2014
Source: BMC Infect Dis. 2016 Nov 28;16:714. doi: 10.1186/s12879-016-2060-7 (PMC5126865; doi:10.1186/s12879-016-2060-7)
Supplement: Additional file 1: Table S1. — Summary of Studies Protocol’s inclusion and exclusion criteria. (DOC 34 kb) [file 12879_2016_2060_MOESM1_ESM.doc]

Table S1. Summary of Studies Protocol’s inclusion and exclusion criteria

| **Study Protocols** | **Inclusion Criteria** | **Exclusion Criteria** | **Number of confirmed Tuberculosis Patients included in the analysis n (%)** |
| --- | --- | --- | --- |
| **Study # 1** | (A) HIV negative; BCG vaccinated with TST <15mm; (B) HIV positive and BCG vaccinated with TST < 5mm; (C) HIV negative and pulmonary MTB; (D) HIV negative and Disseminated MTB; (E) HIV positive and pulmonary MTB; (F) HIV positive and Disseminated MTB | Age < 18 years, and/or Hg <7.5g/dl, and/or Latent MTB infection, and/or **past history of treated MTB infection** | **67 (19.5)** |
| **Study # 2** | (A) Positive smear results of either Ziehl Neelsen or Auramine Rhodamine staining **with or without history of MTB infection**; (B) Age of 18 years old or greater | Anemia (Hg< 10g/dl), and/or age < 18 years old | **115 (33.5)** |
| **Study # 3** | (A) Newly positive smear results of either Zielh Nelseen or Auramine Rhodamine staining; (B) Age of 18 years old or greater; (C) HIV negative | Age < 18 years, and/or **previous history of MTB infection**, and/or HIV positive | **42 (12.3)** |
| **Study # 4** | (A) Positive smear results of either Ziehl Neelsen or Auramine Rhodamine staining, and any clinical symptoms of abnormal findings compatible with active TB on a previously performed Chest X-Ray; (B) Age of 12 years old or greater | Negative sputum smear result, and/or age <12 years old | **97 (28.3)** |
| **Study # 5** | (A) Newly diagnosed Tuberculosis patients, (B) age > 18 years old; (C) Hemoglobin > 8g/dl | Patients with **history of MTB infection**, and/or Anemia (Hg< 10g/dl), and/or age < 18 years old | **22 (6.4)** |
| **Total number of confirmed TB patients from studies excluding retreatment TB patients** | | | **131 (38.2)** |
| **Total number of confirmed TB patients from studies including retreatment TB patients** | | | **212 (61.8)** |
| **Total confirmed TB patients** | | | **343 (100)** |

From 2006 and 2014, participants from all Bamako referral health centers were referred by their physicians to the study team and were consecutively screened for eligibility and enrollment.

HIV= Human Immunodeficiency virus; BCG= Bacille Calmette and Guerin; TST= Tuberculin skin test; MTB= *Mycobacterium tuberculosis*; Hg= Hemoglobin
